# Differential responses of soil nematode community to pig manure application levels in Ferric Acrisols

**DOI:** 10.1038/srep35334

**Published:** 2016-10-13

**Authors:** Yi-Ru Yang, Xiao-Gang Li, Zhi-Gao Zhou, Tao-Lin Zhang, Xing-Xiang Wang

**Affiliations:** 1 Key Laboratory of Soil Environment and Pollution Remediation, Institute of Soil Science, Chinese Academy of Sciences, Nanjing, 210008, China; 2 University of the Chinese Academy of Sciences, Beijing, 100049, China; 3 Ecological Experimental Station of Red Soil, Chinese Academy of Sciences, Yingtan, 335211, China

## Abstract

Excessive pig manure application probably degrades arable soil quality in some intensive pig farming areas. The responses of the nematode community to dosages of pig manure were investigated in Ferric Acrisols under 3-season peanut monoculture. Varying dosages of manure (1.75, 3.5, 7, 14 and 28 t·ha^−1^·yr^−1^) in combination with chemical fertilizer were applied to field plots, and chemical fertilizer alone was also applied as a control. With increasing manure application, the abundance of bacterivores and omnivores-predators increased, the abundance of plant parasites decreased, and fungivores abundance exhibited hump-shaped variation. Simpson diversity index and plant parasite index/maturity index of the nematode communities increased to a maximum level at a manure application rate of 3.5 t·ha^−1^·yr^−1^ and then sharply decreased. The changes in the soil nematode community were further determined to be correlated with chemical properties; available phosphorus had the strongest quadratic correlation with the two indices, implying that available phosphorus had a better indicative effect than other soil properties to nematode community. Available phosphorus in soil was deduced from 49 to 64 mg·kg^−1^ with the best nematode communities. Our results emphasized the importance of regular applications of manure in agriculture field to balance nematode diversity and build healthy agro-ecosystems.

Intensive breeding has become an irreversible trend. Many moderate and small-scale pig farms are widely distributed in the hilly red soil regions of subtropical China, causing heavier environmental burdens on local areas. Returning raw or composed manure to agricultural fields is the principal way of recycling livestock waste, which is often considered a useful addition to maintain soil organic matter and consequently improve soil structure^1^,^2^. However, excessive and repeated manure application can cause nitrogen and phosphorus accumulation in the soil and deteriorate soil quality^3^. Therefore, defining appropriate rates of manure application to local soil is imperative to maintain healthy agro-ecosystems.

Nematodes are promising candidates for bioindicators of soil quality that contribute to the decomposition of soil organic matter and nutrient cycling^4^ and occupy central positions in the soil food web^5^. Nematode abundance and community composition are affected by fertilization, soil properties, vegetation and length of cultivation^6^,^7^,^8^,^9^. Maturity index, plant parasite index and structure index etc. are used to indicate nematode community condition in the context of soil quality in agricultural systems^10^.

Generally, increases in fungivores and plant parasite nematodes^11^,^12^ and decreases in omnivores-predators and in the structure index (*SI*)^13^,^14^ under chemical fertilization addition indicate deterioration of soil quality^15^. Manure application generally improves soil quality and favours greater soil food web complexity. Appropriate manure addition in soil increased the abundance of bacterivores and fungivores and decreased plant parasites when compared with chemical fertilizer^16^,^17^,^18^. Omnivores-predators clearly increased in abundance with high rates of manure application^19^, whereas they remained constant with low-level application of manure^17^. However, various responses of nematode communities to manure application depend on the dosages at which it is applied to soils because of the sensitivity of soil nematodes to additives. Thus, dose-effects of manure on the soil nematode community require further investigation under multi-level manure application in practical cultivation systems.

The changes in both nematode abundance and community composition are related to the chemical properties of soils under manure application. For example, manure application increased soil pH and concentrations of organic carbon, nitrogen and phosphorus^20^,^21^, which had positive effect on the abundances of total nematodes and bacterivores and the diversity index of the nematode community^11^,^12^. Nonetheless, quantitative relationships of variable chemical properties and the development of nematode community under varying dosages of manure application were seldom concerned. We hypothesize that high level of manure application degrade soil nematode community by the tremendous changes of soil chemical properties. A better understanding of the chemical properties and quantification of their effects on the soil nematode community, thus, would allow the determination of the mechanical impact of manure application on soil quality and the limitations of that application.

Ferric Acrisol, one of the dominant soils in the hilly red soil regions of subtropical China, supports substantial agricultural products for China, but its quality needs to be improved because it is acidic and low in organic matter. The objectives of this study were to investigate the responses of the soil nematode community to dosages of pig manure applied to Ferric Acrisols and the relationships between the changes in the nematode community and the soil chemical properties and to determine appropriate dosage of manure for Ferric Acrisols based on the quantitative relations between the nematode community and soil chemical properties.

## Results

### The density and variation of soil nematodes community

Manure application significantly affected the density of total nematodes (*F* = 19.166, *p* < 0.001). The soil nematode density increased with increasing application of manure. The total nematode density ranged from 205.0 individuals in the treatment with chemical fertilizer alone (CK) to 669.5 individuals in the treatment with 28 t·ha^−1^·yr^−1^ of manure application (Table 1).

A total of twenty-two nematode genera were identified across all samples. In the chemical fertilizer alone treatment, *Tylenchus* was the most abundant nematode genus, whereas *Eucephalobus*, *Aporcelaimus* and *Prodorylaimus* were the dominant genera in the manure treatments. *Prismatolaimus*, *Criconemoides*, *Mononchus* and *Mylonchulus* were not identified in CK but appeared in the manure treatments. Furthermore, with increasing quantities of manure applied, the density of *Eucephalobus* and *Prodorylaimus* sharply increased, whereas the density of *Pratylenchus* sharply declined. *Filenchus* density increased with manure application, reached its maximum at the manure rate of 3.5 t·ha^−1^·yr^−1^ (P2), and then declined gradually*. Aporcelaimus* increased with increasing manure application, but its density decreased significantly with the highest rate of manure application (28 t·ha^−1^·yr^−1^, P5).

### The distribution of the nematode trophic groups

Among the four trophic groups, the relative abundances of plant parasites and fungivores differed significantly across all the treatments. Manure effects were also observed on the relative abundances of bacterivores and omnivores-predators. In the chemical fertilizer alone treatment (CK), plant parasites accounted for 42.2% of total soil nematodes and was the dominant trophic group (Table 1). In the manure treatments, bacterivores and omnivores-predators became the dominant trophic groups, reaching 46.4% and 44.6% of total abundance in the highest manure treatment (28 t·ha^−1^·yr^−1^, P5), respectively. The relative abundances of bacterivores increased significantly (*F* = 9.965, *p* < 0.001), whereas the relative abundance of plant parasites decreased significantly with increasing manure application (*F* = 35.273, *p* < 0.001). The abundance of fungivores varied significantly among all manure treatments (*F* = 19.880, *p* < 0.001). The abundance of fungivores reached its maximum at the manure rate of 3.5 t·ha^−1^·yr^−1^ (P2) and then declined gradually with increasing manure application. The relative abundance of omnivores-predators increased gradually as the rates of manure application increased to 14 t·ha^−1^·yr^−1^ (P4) and then decreased slightly at the manure rate of 28 t·ha^−1^·yr^−1^ (P5).

### Diversity and the ecological indices of soil nematode communities

**Figure 1:**
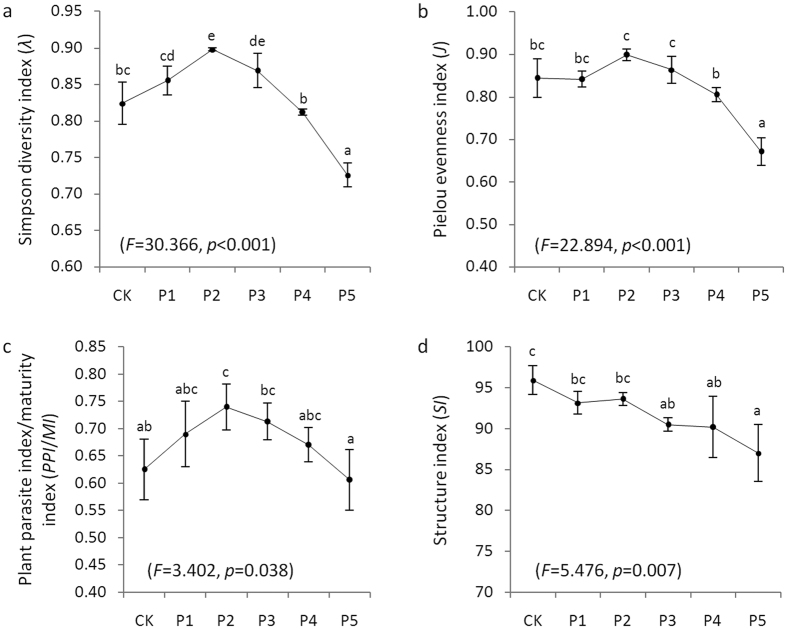
Diversity (**a,b**) and the ecological indices (**c,d**) of soil nematode communities under different fertilizer treatments. Error bars represent the standard errors. Different lowercase letters indicate significant differences among treatments (*p* < 0.05). CK: chemical fertilizer alone; and P1–P5: chemical fertilizer with the addition of pig manure at rates of 1.75, 3.5, 7, 14 and 28 t·ha^−1^·yr^−1^, respectively.

Significant effects of manure application were demonstrated by the Simpson diversity index (*λ*, *F* = 30.366, *p* < 0.001) and the Pielou evenness index (*J*, *F* = 22.894, *p* < 0.001) (Fig. 1a,b). The value of *λ* increased significantly with increasing manure application, reaching the maximum value at the manure rate of 3.5 t·ha^−1^·yr^−1^ (P2), and then sharply decreased, while the value of *J* showed slight variation at lower dosages of manure application (<3.5 t·ha^−1^·yr^−1^). The lowest mean values of these indices were observed in the highest manure treatment (P5) and were even lower than that of the chemical fertilizer alone treatment (CK).

Manure application also significantly influenced the plant parasite index/maturity index (*PPI*/*MI*, *F* = 3.402, *p* = 0.038, Fig. 1c). The *PPI*/*MI* ratio increased significantly with increasing manure application, reached its maximum at the manure rate of 3.5 t·ha^−1^·yr^−1^ (P2), and then decreased obviously. The *SI* of the nematode community decreased with increasing manure application (*F* = 5.476, *p* = 0.007, Fig. 1d), but there was no obvious difference between the treatments with lower dosages of manure application (<3.5 t·ha^−1^·yr^−1^), and it was also no significant change between the treatments with higher dosages of manure application (>3.5 t·ha^−1^·yr^−1^).

### Relationship between nematode communities and soil properties

**Figure 2:**
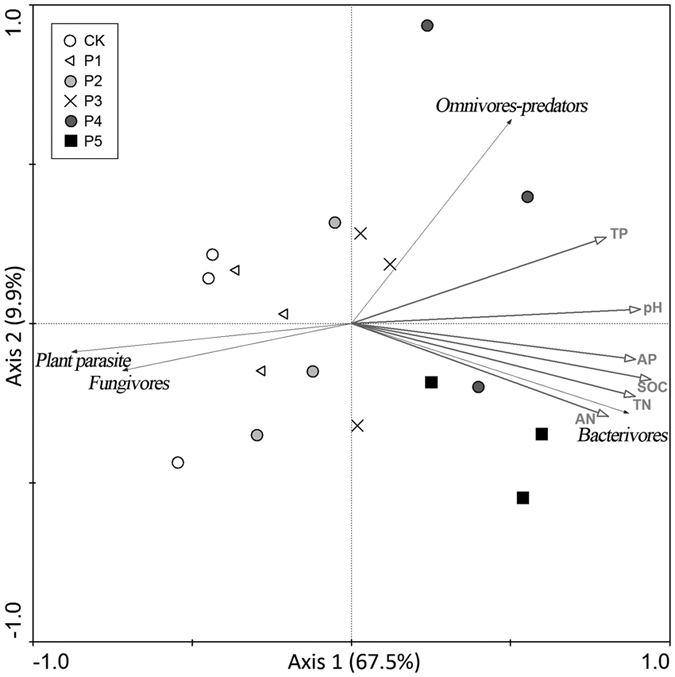
Redundancy analysis (RDA) between nematodes and soil properties. RDA results were summarized in a bi-dimensional plot. The eigenvalues of the first and the second axes were 0.675 and 0.099, respectively. The species-environment correlations for both the first and the second axes were 0.932 and 0.744, respectively. The first axis explained 67.5% of the cumulative variance in species data and 85.9% in species-environment relationships. CK: chemical fertilizer alone; P1–P5: chemical fertilizer with the addition of pig manure at rates of 1.75, 3.5, 7, 14 and 28 t·ha^−1^·yr^−1^, respectively; SOC: soil organic carbon; AN: alkaline nitrogen; AP: available phosphorus; TN: total nitrogen; TP: total phosphorus; and pH: pH value.

The ordination diagram was based on redundancy analysis (RDA) of the nematode community composition and the soil property data for all treatments (Fig. 2 and Supplementary Table S1). As reflected by the Axis 1, no variation was evident in nematode community composition among the chemical fertilizer alone treatment (CK) and the two lower manure treatments (P1 and P2), but noticeable transformations to the positive direction of Axis 1 of nematode community composition occurred with greater than 3.5 t·ha^−1^·yr^−1^ manure application. Nematode community composition continued to vary significantly under the high manure application rates (P3, P4 and P5), as reflected in the Axis 2. The first axis was positively related to soil organic carbon (SOC), pH, available phosphorus (AP), total nitrogen (TN), total phosphorus (TP) and alkaline nitrogen (AN), indicating that these soil properties drove the variation in nematode community composition. The abundance of bacterivores was positively correlated with SOC, TN and AP. The abundance of fungivores was negatively correlated with pH and TN. The abundance of plant parasites was negatively correlated with pH and SOC. The abundance of omnivores-predators was positively correlated with TP and pH. Pearson’s correlation analysis further confirmed the significant responses of bacterivores, fungivores and plant parasites to the above mentioned soil properties (Table 2).

**Figure 3:**
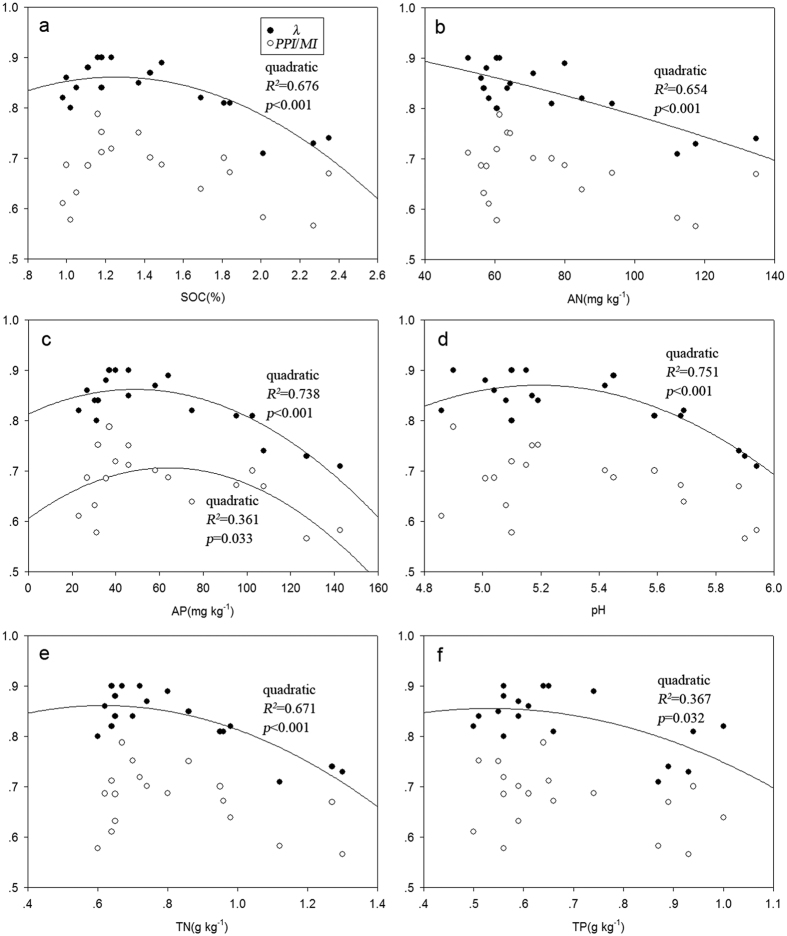
Relationships between Simpson diversity index (*λ*), plant parasite index/maturity index (*PPI*/*MI*) and soil properties (SOC (**a**), AN (**b**), AP (**c**), pH (**d**), TN (**e**), and TP (**f**)) (n = 18). Each data point corresponds to the value of one manure treatment. Quadratic regressions best fit the data. Regression coefficients are reported if *p* < 0.05.

Diversity and the ecological indices can reflect useful information on the dynamics of nematode community. Since indices of *λ* and *PPI*/*MI* of nematode community showed more sensitive to manure than *J* and *SI*, they were selected to conduct curve fitting analysis with soil properties. It demonstrated that the index of *λ* had the strongest quadratic correlation with pH and AP, followed by SOC, TN, AN and TP (*p* < 0.05, Fig. 3a–f). However, no correlation between *PPI*/*MI* and either SOC, TN, AN, pH, or TP was found. *PPI*/*MI* had a quadratic correlation only with AP (*R*^*2*^ = 0.361, *p* = 0.033). These indicated that AP had a better indicative effect than other soil properties to nematode community. The indices of *λ* and *PPI*/*MI* of soil nematodes community reached the highest value when the content of available phosphorus in soil were at 49 and 64 mg·kg^−1^ obtained by the parabolic equation respectively.

## Discussion

The density of total soil nematodes increased significantly with manure addition at application rates of up to 28 t·ha^−1^·yr^−1^, indicating that manure persistently provides food for nematode reproduction^19^,^22^. However, *Pratylenchus*, a dominant genus in plant parasites, decreased significantly in abundance with increasing manure application, which could account for decreasing plant parasites abundance with increasing manure application^23^,^24^,^25^. *Eucephalobus*, *Aporcelaimus* and *Prodorylaimus* contributed most to the variation across manure treatments, which caused substantial increases in abundance of both bacterivores and omnivores-predators. Populations of microbivorous nematodes increase rapidly with the addition of organic fertilizer to soil^26^. In the present study, the abundance of fungivores appeared to decrease suddenly when the rates of manure application exceeded 3.5 t·ha^−1^·yr^−1^, which was in accordance with the results of Jiang *et al*., who found higher abundance of fungivores with lower rates of manure application than with higher rates^17^. Our results combined with this previous study indicate that higher manure application could reduce fungivores growth in soil. Conversely, the abundance of bacterivores increased persistently with increasing manure application, which suggested that the responses of bacterivores and fungivores to manure addition varied with the increasing dosages of manure. Furthermore, greater density of omnivores-predators may have top down pressure on target prey such as fungivores, whereas bacterivores exhibited bottom-up control from the abundant organic manure food source. As a whole, the composition of the nematode community gradually developed from dominance of plant parasites to both bacterivores and omnivores-predators with increasing of manure application rates.

Manure application has generally been found to increase nematode diversity indices^11^,^17^. However, we found that Simpson diversity and Pielou evenness indices were reduced significantly by high manure application (>3.5 t·ha^−1^·yr^−1^). Higher manure inputs appear to favor a few highly competitive species, which become dominant and suppress the growth and reproduction of other species, thereby causing a loss of biodiversity^27^,^28^. For example, the abundance of *Eucephalobus*, *Aporcelaimus* and *Prodorylaimus* was raised significantly at the expense of certain nematode genera, i.e., *Pratylenchus*, *Filenchus*, *Prismatolaimus*, *Criconemoides* and *Mononchus* which were extirpated from the soil of high manure application. A higher *PPI*/*MI* ratio of nematode community indicates ecosystem enrichment and restoration^29^, and higher *SI* suggests a longer food chain^30^. The hump-shaped variation in the *PPI*/*MI* ratio and the decrease in *SI* occurred with increasing rates of manure application, which implied that higher manure application created a poor-structured and simple nematode community, due to the mushroom of bacterivores^31^. These results confirmed our hypothesis that excessive manure application could pose negative pressure on soil nematode community, resulting in the simple soil food web. It is suggested that manure application should be kept at moderate levels to improve the nematode community.

Noticeable transformations of nematode community composition were found for the substantial changes in soil chemical properties under higher manure application. The large increase in bacterivore abundance was most strongly related to SOC, TN and AP, implying that the availability of organic matter and nutrient contents can positively promote increases in soil bacteria and bacterivores^11^,^32^. The changes in fungivores and omnivores-predators were correlated significantly with pH and TP/TN, which demonstrated that the raised pH and nutrient contents in high manure application enhanced the availability of food resources for omnivores-predators and, in turn, placed top down pressure on fungivores^22^. The decrease in plant parasites abundance was primarily associated with the raised pH with the increasing manure application. Oka *et al*. also demonstrated that increasing soil pH from acidic to approximately neutral conditions was effectively nematicidal and possibly controlled plant parasites^33^. It appears that regulating the pig manure input to acidic soils (e.g., Ferric Acrisol) might help to control the plant-feeding nematode populations by raising soil pH. However, in the meantime to reduce the plant parasites, a trade-off of manure rates must be considered to maintain a balanced nematode community assemblage^28^.

The index of *λ* showed parabolic relations with soil properties, indicated that proper nutrients promoted nematode diversity, whereas excess nutrients in soil under higher manure application could result in salt toxicity that could harm soil organisms^34^ and lead to the degradation of nematode community^35^,^36^. The variation of nematode community was related to all these chemical properties and might be the result of a combination of all these environmental factors. AP had the most sensitive response to manure application among the chemical properties, and it accumulated rapidly in soils with increasing manure application^37^. Compared with other soil properties, AP had better goodness-of-fit relationships not only with Simpson diversity index but also with plant parasite index/maturity index, suggesting that AP had a better indicative effect than other soil properties to characterize soil nematode community.

Based on the changes in the soil nematode community indices (*λ* and *PPI*/*MI*), optimal AP content in Ferric Acrisols ranges from 49 to 64 mg·kg^−1^, which is similar to the results of Sims who demonstrated the agronomic thresholds of AP ranged from 31 to 55 mg·kg^−1 ^38. The similar critical value indicated that the content of AP in the soil could be an indicator of soil nematode community. However, the corresponding mechanisms of the nematodes under phosphorus stress have not been demonstrated clearly^39^, and need systematic investigation in the future.

In summary, our results revealed differential responses of nematodes to varying dosages of manure application. Low dosages of manure application increased the diversity of nematode community, whereas high dosages of manure application lead to explosive growth of bacterivores and significantly decreased the community diversity. The results highlighted that excessive application of nutrients through manure addition might be inefficient and of no benefit to the soil food web. Soil nematodes were directly contacted with the changes of soil conditions, and available phosphorus had a better indicative effect to soil nematode community change than other chemical properties. It was emphasized that rational fertilizer regime including regular applications of manure would be advocated to balance nematode diversity and build healthy agro-ecosystems.

## Materials and Methods

### Experimental design

The experiment was conducted at the Ecological Experimental Station of Red Soil, Chinese Academy of Sciences in Yujiang County, Jiangxi, China (28°15′20″N, 116°55′30″E). The climate is typical subtropical monsoon. For the last 50 years, the annual mean temperature is 17.6 °C, and the annual precipitation is 1795 mm, with distinct wet (from March to June) and dry seasons (from July to February). The annual mean precipitation was 1776 mm during the experiments (2013–2015).

The experimental plots were established in January 2013 on a local upland with an 8 degree slope. The soil was developed from Quaternary Red Clay (Udic Ferralsols in Chinese Soil Taxonomy and Ferric Acrisols in the FAO classification system) with an SOC concentration of 5.16 g·kg^−1^, AN 59.50 mg·kg^−1^, AP 28.34 mg·kg^−1^, and with pH 4.90. The plots were randomly distributed with three replications. The treatments included 1) chemical fertilizer alone (CK) applied at 50 kg N·ha^−1^·yr^−1^ of nitrogen fertilizer (urea), 20 kg P·ha^−1^·yr^−1^ of phosphorus fertilizer (calcium magnesium phosphate fertilizer), and 55 K·ha^−1^·yr^−1^ of potassium fertilizer (potassium chloride fertilizer), which was half the local conventional inorganic fertilizer application rates for Ferric Acrisols; 2) on the basis of the chemical fertilizer of CK, varying dosages of pig manure at rates of 1.75, 3.5, 7, 14 and 28 t·ha^−1^·yr^−1^ (dry weight) were extra-applied respectively, and were coded as P1, P2, P3, P4, and P5. The pig manure was collected from pig farms near the experimental station and then composted for at least 3 months before application, with a moisture content of approximately 60%. The manure had average total nitrogen, total phosphorus and total potassium concentrations of 22.7, 11.4 and 18.8 g·kg^−1^ (dry matter), respectively.

### Crop planting and soil sampling

Peanuts (*Arachis hypogaea* L.) were planted in April. First, manure and chemical fertilizer were manually broadcast across the soil surface, which was ploughed at a depth of 0–15 cm. Then, plots were ridged equally altitude with 45 cm row spacing and were holed 5 cm in depth and 17 cm plant spacing on the ridges. Two peanut seeds were sowed in each hole and then covered with surface soil. Field management was in accordance with local farming practices, with the exception of hand weeding (Table 3). Peanuts were harvested in August from each plot. The pods, straws and roots were cleaned and weighed separately. From September to the following March, all plots were fallow.

Soil was sampled at a depth of 0–15 cm in each plot before peanut harvesting in August 2015 using an auger with a 3 cm internal diameter. Four random cores per plot were taken from between the peanut plants on the ridges, and the cores were then composted as one sample. After removing stones and larger roots, the soil was divided into two aliquots. One aliquot was immediately taken to the laboratory for extraction and identification of soil nematodes, and the other was air-dried and passed through soil sieves (2 mm and 0.149 mm) for the analysis of soil chemical properties.

### Soil chemical analysis

Soil pH was measured in 1:2.5 (w/v) soil-to-water ratio solutions using a glass electrode pH metre. SOC was determined using the potassium dichromate oxidation method. AN was analysed via the alkaline hydrolysis diffusion method. AP was extracted with 0.5 M NaHCO_3_ and determined via the colorimetric method. TN was determined via the oxidation of sulfuric acid and the semi-micro Kjeldahl nitrogen method. TP was analysed by hydrofluoric acid and perchloric acid digestion and determined via the colorimetric method. To measure soil water content, fresh soil samples were dried at 105 °C for 6 h^40^.

### Nematode extraction and identification

Soil nematodes were extracted by elutriation from 100 g composite fresh soil samples using the sugar flotation and centrifugation method^41^. The nematodes were heat-killed at 60 °C and then preserved in triethanol amine formaldehyde (TAF) solution. The total nematodes were determined using the direct count method under a microscope and were expressed per 100 g dry soil after soil water content was measured. After fixation, 100 randomly selected individuals per sample were identified to genus under a microscope (Eclipse E200, Nikon Corporation, Tokyo, Japan). The nematode genera were classified into the following four trophic groups based on morphological characteristics, feeding habits and life cycle: bacterivores, fungivores, plant parasites and omnivores-predators^26^,^42^,^43^. Nematode genera were also assigned to ecological guilds as determined by the trophic behaviour of the nematode and by its ecological life strategy on the colonizer-persister (*c-p*) scale (1–5)^30^,^42^,^44^. All nematodes were identified to genus, trophic group and ecological guild when the sample contained fewer than 100 individuals.

### Nematode community diversity and the ecological indices

Diversity and the ecological indices of the soil nematode communities were characterized by calculating the following specific indices:

1. Simpson diversity index^45^, *λ* = 1-∑*pi*^2^;
2. Pielou evenness index^46^, *J* = (-∑*pi* × ln*pi*)/ln*S*, where ‘*pi*’ is the proportion of the individuals in “*i*th” group in the nematode community, and ‘*S*’ is the total number of nematode genera in the community.
3. Maturity index, *MI* = ∑*c-pi* × *f(i)*, where *c-pi* is the *c-p* value of free-living nematode genus *i*, and *f(i)* is the frequency of genus *i* of the total number of free-living nematodes in a sample. Free-living nematodes include all nematode genera except plant parasites; plant parasite index, *PPI* = ∑*c-pi* × *f(i)*, where *c-pi* is the *c-p* value of plant parasitic nematode genus *i*, and *f(i)* is the frequency of genus *i* of the total number of plant-parasitic nematodes in a sample. The ratio of *PPI*/*MI* was used to evaluate the nematode communities^47^.
4. Structure index^30^, *SI* = 100 × s/(s + b), where ‘b’ is the basal food web component, b = 0.8 × (Ba_2_ + Fu_2_); and ‘s’ is the structure component, s = 0.8 × Pr_2_ + 1.8 × (Ba_3_ + Fu_3_ + Om_3_ + Pr_3_) + 3.2 × (Ba_4_ + Fu_4_ + Om_4_ + Pr_4_) + 5 × (Ba_5_ + Fu_5_ + Om_5_ + Pr_5_); Ba: bacterivores; Fu: fungivores; Om: omnivores; Pr: predators; and digital in the lower right corner is the *c-p* value of nematode genus.

### Data analysis

A one-way analysis of variance (ANOVA) was performed to analyse the impacts of fertilization on the total nematode density, nematode trophic groups, diversity and the ecological indices of the nematode communities across all treatments. The Duncan multiple comparison test was then used to find statistically significant differences among the different treatments. Differences at *p* < 0.05 were considered statistically significant. Linear correlations between nematode community composition and soil properties were quantified using Pearson’s correlation coefficients. All of these analyses were performed using SPSS 16.0 (SPSS Inc., Chicago, IL).

Multivariate analysis was used to explore the responses of nematode communities to the treatments, with soil properties as environmental variables using CANOCO version 4.5^48^. Because linear responses of nematodes to environmental gradients were found in advance by detrended correspondence analysis of categories indicating a maximum gradient length below 4 for each category (Lengths of gradient: 1.236), redundancy analysis (RDA) was conducted rather than canonical correspondence analysis. Relative abundance of nematode trophic groups was used as species data. Among the soil properties, SOC, pH, AP, TN, TP and AN were used as environment data. The RDA result was shown in a bi-dimensional plot. Curve fitting was used to examine the relationships between the indices of nematode communities (*λ* and *PPI*/*MI*) and the soil properties across all treatments, and scatter plots were drawn using SigmaPlot (Systat Software Inc., San Jose, CA).

## Additional Information

**How to cite this article**: Yang, Y.-R. *et al*. Differential responses of soil nematode community to pig manure application levels in Ferric Acrisols. *Sci. Rep.* **6**, 35334; doi: 10.1038/srep35334 (2016).

## Supplementary Material

Supplementary Information

## Figures and Tables

**Table 1:** Mean density (individuals per 100 g dry soil, n = 3) of soil nematodes and the relative abundance of nematode trophic groups under different fertilizer treatments.

Genus	*c-p*	CK	P1	P2	P3	P4	P5
Bacterivores	Density	24.7 ± 16.8a*	53.0 ± 14.8a	83.9 ± 19.0a	164.2 ± 64.9b	190.5 ± 62.3b	309.3 ± 53.1c
	Relative abundance (%)	12.1% ± 7.5%a	16.8% ± 0.8%a	19.7% ± 2.5%a	33.1% ± 8.2%b	37.1% ± 10.4%b	46.4% ± 9.1%b
*Protorhabditis*	Ba_1_	2.9 ± 2.9a	6.6 ± 6.6a	29.1 ± 25.6a	30.7 ± 37.7a	29.7 ± 29.7a	11.7 ± 6.6a
*Rhabditis*	Ba_1_	8.7 ± 10.5a	15.8 ± 4.0ab	18.5 ± 9.1ab	8.3 ± 0.1a	14.9 ± 14.9ab	44.9 ± 39.3b
*Cephalobus*	Ba_2_	0a	3.3 ± 3.3b	0a	0a	0a	0a
*Eucephalobus*	Ba_2_	13.1 ± 7.3a	17.9 ± 6.1a	21.1 ± 7.3a	112.6 ± 36.7b	145.9 ± 47.4b	250.0 ± 25.3c
*Prismatolaimus*	Ba_2_	0a	9.3 ± 2.7b	11.3 ± 4.4b	12.6 ± 4.3b	0a	2.8 ± 2.8a
*Alaimus*	Ba_4_	0a	0a	3.9 ± 3.9b	0a	0a	0a
Fungivores	Density	21.8 ± 1.9a	29.0 ± 9.8ab	70.1 ± 16.1c	40.9 ± 3.8ab	21.3 ± 4.3a	20.5 ± 3.2a
	Relative abundance (%)	10.9% ± 2.5%b	9.1% ± 1.6%b	16.4% ± 2.1%c	8.7% ± 2.5%b	4.3% ± 1.3%a	3.1% ± 0.5%a
*Aphelenchus*	Fu_2_	2.7 ± 4.7a	0a	0a	0a	0a	0a
*Filenchus*	Fu_2_	4.1 ± 4.1a	19.9 ± 8.1b	49.3 ± 9.9c	20.1 ± 3.6b	17.0 ± 0b	13.8 ± 3.1ab
*Diphtherophora*	Fu_3_	2.9 ± 2.9b	0a	0a	0a	0a	0a
*Tylencholaimus*	Fu_4_	12.1 ± 6.4ab	9.1 ± 3.8a	20.9 ± 6.7b	20.8 ± 3.9b	4.3 ± 4.3a	6.7 ± 5.8a
Plant parasites	Density	85.1 ± 8.4bc	120.6 ± 52.5c	77.0 ± 22.0abc	55.0 ± 15.4ab	32.8 ± 9.7a	40.5 ± 23.7ab
	Relative abundance (%)	42.2% ± 7.2%c	37.1% ± 6.2%c	18.0% ± 3.7%b	11.4% ± 2.3%ab	6.6% ± 2.5%a	6.0% ± 3.2%a
*Tylenchus*	Pp_2_	48.1 ± 7.4a	72.1 ± 60.3a	30.5 ± 16.3a	28.7 ± 5.1a	22.8 ± 11.2a	40.5 ± 23.7a
*Criconemoides*	Pp_3_	0a	2.0 ± 2.0ab	6.3 ± 1.5c	4.7 ± 4.7bc	0a	0a
*Pratylenchus*	Pp_3_	36.9 ± 9.2c	46.5 ± 6.8c	40.2 ± 7.6c	21.6 ± 12.2b	10.0 ± 1.5ab	0a
Omnivores-predators	Density	73.4 ± 30.8a	112.5 ± 16.0a	191.7 ± 12.9b	224.8 ± 28.1bc	267.0 ± 70.2c	299.2 ± 51.5c
	Relative abundance (%)	34.8% ± 10.0%a	36.9% ± 6.5%ab	45.9% ± 8.0%ab	46.9% ± 5.2%ab	52.1% ± 12.3%b	44.6% ± 5.6%ab
*Dorylaimoides*	Om_4_	0a	0a	10.3 ± 10.3b	2.4 ± 2.4a	0a	0a
*Eudorylaimus*	Om_4_	5.8 ± 5.8ab	7.9 ± 3.9b	5.8 ± 1.1ab	0a	2.9 ± 2.9ab	5.6 ± 5.6ab
*Microdorylaimus*	Om_4_	0a	0a	0a	0a	0a	4.7 ± 4.7b
*Thorneella*	Om_4_	0a	3.5 ± 3.3b	0a	0a	0a	0a
*Aporcelaimus*	Om_5_	46.7 ± 28.2a	55.6 ± 3.6a	55.8 ± 1.0a	93.9 ± 34.1b	100.5 ± 12.2b	39.4 ± 7.3a
*Mesodorylaimus*	Om_5_	2.9 ± 2.9a	12.6 ± 7.0ab	14.2 ± 14.2ab	28.2 ± 16.9b	23.0 ± 6.0ab	11.1 ± 11.2ab
*Prodorylaimus*	Om_5_	18.0 ± 6.5a	19.0 ± 13.0a	77.3 ± 5.9ab	50.2 ± 8.9ab	95.1 ± 64.3b	223.1 ± 65.8c
*Mylonchulus*	Pr_4_	0a	4.0 ± 4.0ab	2.4 ± 2.4ab	16.5 ± 16.5b	5.8 ± 5.8ab	4.7 ± 4.7ab
*Mononchus*	Pr_4_	0a	9.9 ± 9.9a	25.9 ± 2.5b	33.6 ± 8.9bc	39.7 ± 5.3c	10.7 ± 10.7a
Total	Density	205.0 ± 33.4a	315.1 ± 91.0ab	422.8 ± 45.8bc	484.9 ± 87.5c	511.5 ± 59.1c	669.5 ± 43.9d

^*^The mean and standard error of the density of soil nematodes and the relative abundances of nematode trophic groups (%) under the different fertilizer treatments are shown. Different lowercase letters indicate significant differences among the treatments (*p* < 0.05). CK: chemical fertilizer alone; P1–P5: chemical fertilizer with the addition of pig manure at rates of 1.75, 3.5, 7, 14 and 28 t·ha^−1^·yr^−1^, respectively; *c-p*: colonizer-persister group; Ba: bacterivores; Fu: fungivores; Pp: plant parasites; Om: omnivores; and Pr: predators.

**Table 2:** Pearson’s correlation coefficients between nematode community composition and soil properties (n = 18).

Soil properties	Nematode trophic groups			
	Bacterivores	Fungivores	Plant parasites	Omnivores-predators
SOC	0.879^**^	−0.739^**^	−0.796^**^	0.370
AN	0.802^**^	−0.695^**^	−0.655^**^	0.236
AP	0.819^**^	−0.722^**^	−0.758^**^	0.386
pH	0.794^**^	−0.815^**^	−0.775^**^	0.501^*^
TN	0.854^**^	−0.740^**^	−0.739^**^	0.315
TP	0.626^**^	−0.660^**^	−0.719^**^	0.581^*^

*,**: correlation coefficient is significant at the 0.05 and 0.01 level (2-tailed), respectively. SOC: soil organic carbon; AN: alkaline nitrogen; AP: available phosphorus; TN: total nitrogen; TP: total phosphorus; and pH: pH value.

**Table 3:** Timeline for agricultural practices in the plots during this study (2013–2015).

Agricultural practices	Planting seasons		
	2013	2014	2015
Ploughing, applying manure & fertilizer	14-Apr	06-Apr	13-Apr
Sowing	15-Apr	07-Apr	14-Apr
Ridging	20-Jun	22-Jun	25-Jun
Weeding	04-May	25-Apr	01-May
	19-May	14-May	17-May
	06-Jun	02-Jun	08-Jun
	20-Jun	22-Jun	25-Jun
	11-Jul	08-Jul	09-Jul
	27-Jul	23-Jul	24-Jul
Harvesting	15-Aug	07-Aug	08-Aug
