# Supplementary material for: Differential responses of soil nematode community to pig manure application levels in Ferric Acrisols
Source: Sci Rep. 2016 Oct 13;6:35334. doi: 10.1038/srep35334 (PMC5062124; doi:10.1038/srep35334)
Supplement: Supplementary Information [file srep35334-s1.pdf]

Title:

Differential responses of soil nematode community to pig manure application levels in Ferric Acrisols

Author:

Yi-Ru Yang, Xiao-Gang Li, Zhi-Gao Zhou, Tao-Lin Zhang, Xing-Xiang Wang*

Table S1. Mean concentrations of soil chemical properties under different fertilizer treatments (n=3).

	SOC(%)	AN($mg\cdot kg^{-1}$)	AP($mg\cdot kg^{-1}$)	pH	TN($g\cdot kg^{-1}$)	TP($g\cdot kg^{-1}$)
CK	1.00±0.02a*	58.36±2.24a	27.05±4.01a	5.00±0.12a	0.62±0.02a	0.56±0.05a
P1	1.12±0.06ab	59.36±3.69a	32.64±2.63a	5.09±0.09a	0.67±0.03a	0.55±0.04a
P2	1.19±0.04b	58.11±4.98a	40.89±4.45ab	5.05±0.13a	0.68±0.04a	0.62±0.05a
P3	1.43±0.06c	71.83±7.88a	55.95±9.30b	5.35±0.15b	0.80±0.06b	0.63±0.10a
P4	1.78±0.08d	84.93±8.60b	90.91±14.35c	5.65±0.06c	0.96±0.02c	0.87±0.18b
P5	2.21±0.18e	121.47±11.74c	125.87±17.53d	5.91±0.03d	1.23±0.10d	0.89±0.03b

*The mean and standard error of soil chemical properties under the different fertilizer treatments are shown. Different lowercase letters indicate significant differences among the treatments ($p<0.05$). CK: chemical fertilizer alone; P1-P5: chemical fertilizer with the addition of pig manure at rates of 1.75, 3.5, 7, 14 and 28 $t\cdot ha^{-1}\cdot yr^{-1}$, respectively; SOC: soil organic carbon; AN: alkaline nitrogen; AP: available phosphorus; TN: total nitrogen; TP: total phosphorus; and pH: pH value.
